# Improved method of step length estimation based on inverted pendulum model

**DOI:** 10.1177/1550147717702914

**Published:** 2017-04-10

**Authors:** Qi Zhao, Boxue Zhang, Jingjing Wang, Wenquan Feng, Wenyan Jia, Mingui Sun

**Affiliations:** 1 School of Electronic and Information Engineering, Beihang University, Beijing, China; 2 Department of Neurosurgery, University of Pittsburgh, Pittsburgh, PA, USA; 3 Department of Electrical and Computer Engineering, University of Pittsburgh, Pittsburgh, PA, USA

**Keywords:** Step length, inverted pendulum model, wearable computer, empirical mode decomposition, inertia measurement unit sensors

## Abstract

Step length estimation is an important issue in areas such as gait analysis, sport training, or pedestrian localization. In this article, we estimate the step length of walking using a waist-worn wearable computer named eButton. Motion sensors within this device are used to record body movement from the trunk instead of extremities. Two signal-processing techniques are applied to our algorithm design. The direction cosine matrix transforms vertical acceleration from the device coordinates to the topocentric coordinates. The empirical mode decomposition is used to remove the zero- and first-order skew effects resulting from an integration process. Our experimental results show that our algorithm performs well in step length estimation. The effectiveness of the direction cosine matrix algorithm is improved from 1.69% to 3.56% while the walking speed increased.

## Introduction

Step length estimation is important in a number of applications such as pedestrian navigation,^1–3^ gait analysis,^4^ medical rehabilitation, and sports training. In pedestrian navigation, step length and head orientation together decide the precision of location and navigation. Therefore, promoting precision of the step length estimation is essential for a better pedestrian navigation service. In other fields, such as gait analysis, medical rehabilitation, and sports training, both the step length and the changing rate of step length are important parameters for assessing health or motion intensity. Two popular methods are used to estimate the step length. On one hand, some researches concentrated on the empirical equations between the step length and other parameters such as walking frequency or vertical acceleration.^5–12^ On the other hand, researches estimated step length based on biological model. They used one or two inverted pendulums to model the leg movement.^13–16^ In this way, the problem of how to estimate step length transformed to the problem of how to calculate the vertical distance of each step. And the usual solution is double integration of vertical acceleration, and the key point is how to find the start and the end of the double integration step by step. In this study, we aimed at estimating step length using motion sensors based on inverted pendulum algorithm. Unlike previous model-based studies, we calculated double integration for the whole vertical acceleration, instead of calculating them step by step. In this way, we avoided finding the start and the end integration point step by step, which could easily cause drift errors. The gravity and magnetic components are used to calculate the direction cosine matrix (DCM)^17^ for coordinate transformation. Then, the body movement component is multiplied with the calculated DCM, which transforms the component from the device coordinate to a topocentric coordinate. Furthermore, we used the empirical mode decomposition (EMD) algorithm^18^ to reduce the accumulated noise after each integration, which can separate linear error from a complicated waveform with a lower computational load.

## Related work

Existing methods to estimate the step length can be categorized into two approaches: one is based on empirical relationship and the other one is based on biomechanical models.

### Empirical relationship

The empirical approach uses either linear^5–9^ or non-linear^10–12^ relationship between the step length and other measured parameters. The relationship between the step length and the walking frequency is linear, which is described in equations (1) and (2)^5–9^

(1) $$SL=K_{1}\times f_{\mathrm{step}}+K_{2}$$

(2) $$SL=\alpha \times f_{\mathrm{step}}+\beta \times \nu +\gamma$$

In the equations above, *f_step_* represents the walking frequency. Parameter *K*_1_, *K*_2_ and parameter set (*α*, *β*, *γ*) can be calculated by the training data, and *ν* is the walking velocity.

Several studies concentrated on non-linear empirical relationships between the step length and other parameters. As mentioned in Bylemans et al.,^10^ a step consists of two phases: the swing phase, where the foot is brought forward from behind the other leg, which is then followed by the heel-touch-down phase, where the foot is placed on the ground. During the swing phase, the vertical acceleration will show a negative peak, which is then followed by a positive peak and again a negative peak due to the heel-touch-down phase. The absolute average of all accelerations during the step is calculated to estimate the step size. To adjust the absolute average in proportion to the step, the duration of the step and the difference in peaks are taken into consideration.

The empirical equation (3), based on the work of Bylemans et al.,^10^ is applied to determine the step length

(3) $$SL=0.1\times \sqrt[{2.7}]{\frac{\sum_{i=1}^{N}\left|a_{\mathrm{vert},i}\right|}{N}\cdot \sqrt{\frac{K}{\sqrt{T\cdot a_{\mathrm{peak},\mathrm{diff}}}}}}$$

where *T* is the step duration, *N* is the number of sampling points during the step, *a_vert_*_,_ *_i_* is the vertical acceleration at the *i*th sampling point, *a_peak_*_,_ *_diff_* represents the difference in peaks of the heel-touch-down phase during the step, and *K* is a standard value to adjust the absolute average.

Another non-linear empirical formula is given by^11^

(4) $$SL=0.98\times \sqrt[{3}]{\frac{\sum_{i=1}^{N}\left|a_{\mathrm{foot}.\mathrm{vert},i}\right|}{N}}$$

where *a_foot.vert_*, *_i_* represents the vertical accelerations during the step duration, while the sensor is attached on the ankle.

Finally, a quartic empirical relationship between the step length and the range of the vertical acceleration is proposed by^12^

(5) $$SL=K\times \sqrt[{4}]{a_{\max}-a_{\min}}$$

where *a_max_* and *a_min_*, respectively, represent the maximum value and the minimum value of the vertical acceleration during one step, and *K* is a constant to be determined by calibration.

### Biomechanical model

**Figure 1:**
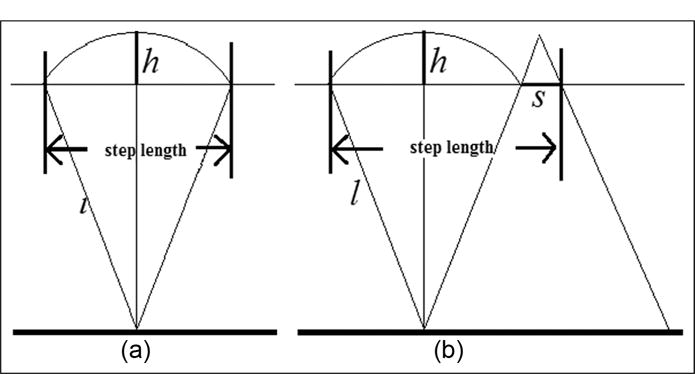
(a) Inverted pendulum model of the leg movement, where *l* is the leg length, *h* is the vertical displacement of the waist, and *h* is calculated by double integration of vertical acceleration. (b) Modified inverted pendulum to model walking, where *S* represents the foot length.

The most popular model-based step length estimation approach utilizes the inverted pendulum model that simulates leg movement while walking,^13^ as shown in Figure 1(a). This model is well applicable to the case that the acceleration is measured at subject’s waist which is close to the body’s center of mass. According to Figure 1(a), we have

(6) $$SL=K\times 2\sqrt{2lh-h^{2}}$$

where *K* is a constant determined by calibration,^13^ *l* is the leg length, *h* is the vertical displacement of the walking step, and *h* is calculated by double integrals of the vertical acceleration.

A more complex biomechanical model contains two inversed pendulums.^14^ The first pendulum models the leg movement during the single-foot stance phase (*L_ss_*), while the second pendulum models the anterior–posterior displacement during the two-foot stance phase (*L_ds_*), as shown in Figure 1(b)

(7) $$SL=L_{ss}+L_{ds}=2\times \sqrt{2lh-h^{2}}+K\cdot S$$

where *L* is the subject’s leg length, *h* is the vertical displacement during one step, *K* is the proportional constant,^15,16^ and *S* is the foot length.

## Methods

There are several shortcomings in the current step length estimation methods. The empirical equation–based methods require large amounts of training data for calibration, and the parameters in empirical equations are different for different subjects. In the model-based methods, the double integrals cause substantial accumulation error, and it is difficult to find the accurate start and end points of the integrals for each step.

Our method utilizes a modified inverted pendulum, as shown in Figure 1(b). And the relationship to describe this model is the same with equation (7). The least squares optimization function is used to find the optimum value of *K*. There are three main differences between our method and previous pendulum model-based method:

1. We add coordinate transformation to obtain vertical acceleration after median and low-pass filtering in data preprocessing.
2. Unlike previous studies which focused on finding a right integrating range in each walking step, we integrate data as a whole without piecewise integrations.
3. We use the EMD technique^18^ to eliminate the accumulation error due to integration.

**Figure 2:**
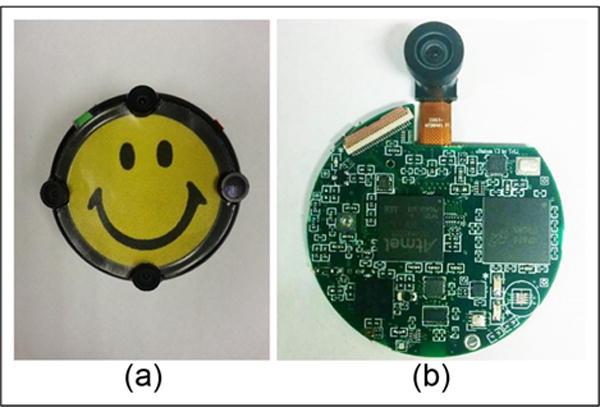
(a) eButton: a wearable computer which contains a microprocessor, four miniature cameras, and an inertia measurement unit (IMU) with a triaxial accelerometer, a triaxial gyroscope, and a triaxial magnetometer. (b) The circuit board of the eButton.

Our device is a wearable computer named eButton which contains a microprocessor, four miniature cameras, and an inertia measurement unit (IMU) with a triaxial accelerometer, a triaxial gyroscope, and triaxial magnetometer, as shown in Figure 2.^19^ The eButton can be worn at different locations on the body, such as the chest and the waist. The motion data acquired by the eButton are either stored within a memory card within the device or transmitted wirelessly to a computer or a mobile device. In this study, we use the vertical component of the waist acceleration to estimate the step length which is one of the most important parameters characterizing walking.

Our method is highlighted as follows. Motion data are acquired by a triaxial accelerometer and a triaxial magnetometer inside the eButton during walking. The acceleration data are filtered by a low-pass filter to separate the gravity component and the body movement component. After low-pass filtering, the gravity and magnetic components are used to calculate the DCM^17^ for coordinate transformation. Then, the body movement component is multiplied with the calculated DCM, which transforms the component from the device coordinate system to the topocentric coordinate system. The topocentric coordinate system is fixed with two of the three axes in the directions of the gravity and magnetic north of the earth, respectively. The third axis follows the right-hand rule. Finally, the transformed body movement component is filtered by a median filter to suppress the noise.

After the data preprocessing, we rely on the vertical acceleration signal to count steps and estimate step length where a double integration procedure is utilized. Because this procedure will inevitably cause accumulative error, we cannot analyze the result directly. Instead, we apply the EMD before step counting and step length estimation, which suppresses the error effectively. In following sections, we describe our method in detail.

### Coordinate transformation

The accelerometer within the eButton detects three axial acceleration components with respect to the coordinate system of the sensor itself. As a result, the vertical axis is not usually perpendicular to the ground, because the wearable device will sway or tilt and the incline angle of the body changes while walking. In contrast, the topocentric coordinate system is fixed with two of the three axes in the directions of the gravity and magnetic north of the earth, respectively, and the third axis follows the right-hand rule. Although the topocentric coordinate system depends on the wearer’s position on the earth, it is still reliable as the walking distance is far shorter than the earth’s radius. Therefore, the topocentric coordinate system is a much stabler and more reliable reference system.

In order to take advantage of the topocentric coordinate system, we apply the DCM^17^ for coordinate transformation on each sampling point. Let Oxyz represent the three axes of the device coordinate system and *i, j, k* represent the unit axial vectors. Likewise, let OXYZ represent the three axes of the topocentric coordinate system and *I, J, K* represent the unit axial vectors. The DCM is given by

(8) $$\mathrm{DCM}=\left[\begin{array}{ccc} \cos\left(I,i\right) & \cos\left(I,j\right) & \cos\left(I,k\right)\\ \cos\left(J,i\right) & \cos\left(J,j\right) & \cos\left(J,k\right)\\ \cos\left(K,i\right) & \cos\left(K,j\right) & \cos\left(K,k\right) \end{array}\right]$$

where (•, •) represents the angle between the two unit axial vectors. Then, any vector ***r*** in the device coordinate system can be transformed to ***R*** in the topocentric coordinate system by

(9) R=DCM×r

Therefore, the key step of the coordinate transformation is to calculate DCM. The detailed procedure has been provided by Premerlani and Bizard.^17^ Briefly, for each sampling point, both the accelerometer and magnetometer are stationary in topocentric coordinate system, but vibrational in the device coordinate system. The DCM can be calculated by the different mappings of a same vector in the two coordinate systems: the directions of the gravity acceleration (9.8 m/s^2^) and the magnetic north of the earth can be determined from the outputs of the accelerometer and the magnetometer, respectively. These two directions define all cosine angles in equation (8). Thus, the DCM is obtained which can be used to evaluate equation (9).

In addition, in this circumstance, the DCM is a time variable matrix for the device coordinate system change with the body movement. So, it is necessary to calculate the DCM for every sampling point and then do coordinate transformation for the data of each sampling point.

### Step length estimation and EMD

As stated previously, we estimate the step length based on the inverted pendulum model. When the person is walking on the ground, the body’s center of mass moves from the back to front of the person, forming the left and right positions of the inverted pendulum (Figure 2(b)). According to this model, the step length can be calculated by equation (10)

(10) $$SL=L_{ss}+L_{ds}=2\times \sqrt{2lh-h^{2}}+1.07\times S$$

where *h* is calculated by double integrations of the vertical acceleration without the gravity component. *l* represents the leg length (leg length is measured from ground to waist when the subject stands straight), and *S* represents the foot length. Equation (10) is the same as equation (7), *K* = 1.07 (section “Find optimum value of K” will introduce how to find the optimum *K* in detail).

To eliminate the accumulation error caused by the baseline drifting and other factors in the integrated result, we propose a novel application of the EMD technique.^18^ As the name “empirical” suggests, this method is based on experience instead of rigorous mathematical deduction. With this method, a complicated signal can be decomposed into a finite and often a small number of intrinsic mode functions (IMF).^18^ We call these modes the IMF components. Each IMF component represents a decomposed waveform of the original data, from high-frequency component to low-frequency component. So, in some occasions, EMD can be used as an effective denoising method.

The decomposition is based on the following assumptions: (1) the signal is supposed to have at least two extrema—one maximum and one minimum; (2) the characteristic time scale is defined by the time lapse between the extrema; and (3) if the data were totally devoid of extrema but contained only inflection points, then it can be differentiated once or more times to reveal the extrema. Final results can be obtained by integration(s) of the components.

The essence of the method is to identify the intrinsic oscillatory modes by their characteristic time scales in the data empirically, and then decompose the data accordingly.

**Figure 3:**
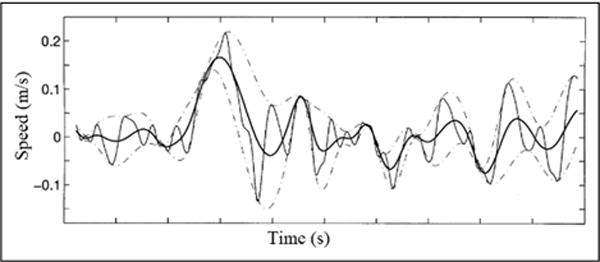
Illustration of the sifting processes: the data are in thin solid line, with the upper and lower envelopes in dot-dashed lines and the mean in thick solid line.

By virtue of the IMF definition, the decomposition method can simply use the envelopes defined by the local maxima and minima separately. Once the extrema are identified, a specific cubic spline line is used to connect all the local maxima to produce the upper envelop. Repeat the procedure for the local minima to produce the lower envelope. The upper and lower envelopes are supposed to cover all the data between them. Their mean is designated as *m*_1_, which is shown in Figure 3, and the difference between the data and *m*_1_ is the first component, *h*_1_

(11) $$X\left(t\right)-m_{1}=h_{1}$$

The sifting process serves two purposes: to eliminate riding waves, and to make the wave-profiles more symmetric. To make this happen, the sifting process has to be repeated *k* times, until *h*_1_*_k_* is an IMF

(12) $$h_{1\left(k-1\right)}-m_{1k}=h_{1k}$$

Then, *c*_1_ is designated as the first IMF component from the data

$$c_{1}=h_{1k}$$

Overall, *c*_1_ should contain the finest scale or the shortest period component of the signal. We need to separate *c*_1_ from the rest of the data by

(13) $$X\left(t\right)-c_{1}=r_{1}$$

Since the residue, *r*_1_, still contains information of longer period components, it is treated as the new data and subjected to the same sifting process as described above. This procedure can be repeated on all the subsequent *r_j_*, and the result is

(14) $$r_{1}-c_{2}=r_{2},\ldots ,r_{n-1}-c_{n}=r_{n}$$

Any of the following predetermined rules can make the sifting process stop: either when the component, *c_n_*, or the residue, *r_n_*, becomes too small that it is less than the predetermined value of substantial consequence, or when *r_n_* becomes a monotonic function from which no more IMF can be extracted.

Thus, we achieved a decomposition of the data into *n*-empirical modes, and a residue *r_n_*, which can be either the mean trend or a constant

(15) $$X\left(t\right)=\sum_{i=1}^{n}c_{i}+r_{n}$$

**Figure 4:**
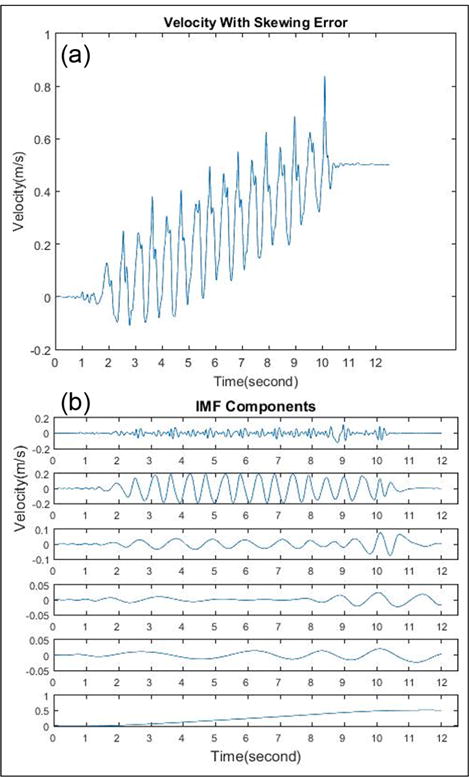
(a) Vertical velocity signal calculated by the direct integration of vertical acceleration from one trial. (b) Six IMF components of vertical velocity signal decomposed with EMD.

**Figure 5:**
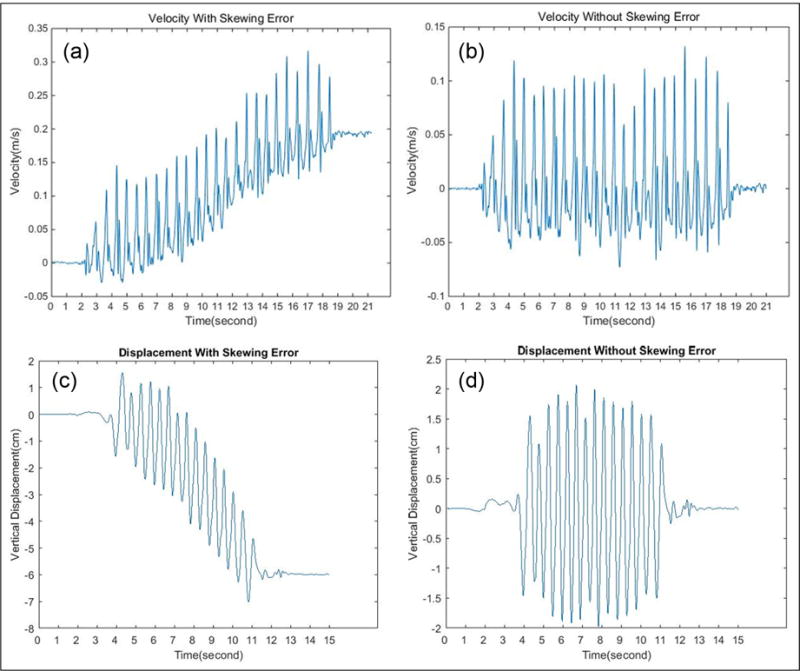
(a) Vertical velocity signal calculated by direct integration of vertical acceleration from one trial. This signal contains accumulation error. (b) Vertical velocity signal after EMD. (c) Vertical displacement signal with accumulation error. (d) Vertical displacement signal after EMD.

Unlike other papers that remove the low-frequency components in the original acceleration signal, we apply EMD on denoising after each integration, because the accumulative error will be more obvious to be removed after integration. Taking advantage of this useful property, we use EMD twice to eliminate baseline drifting after integration. Figure 4(a) shows the vertical velocity signal calculated by the direct integration of vertical acceleration from one trial, and Figure 4(b) shows the six IMF components which are decomposed from the velocity signal by EMD. Our algorithm is given below:

1. Compute the velocity signal by the direct integration of the vertical acceleration signal. An example is shown in Figure 5(a). The result contains the accumulation error so that the output signal cannot be used for vertical displacement calculation directly.
2. Apply the EMD to the vertical velocity signal, discard low-frequency components, and retain the first several IMF components (we experimentally chose first four high-frequency components and discard other low-frequency components). Figure 5(b) shows the sum of the first four IMF components, which represent the velocity signal with the accumulation error suppressed.
3. Calculate the vertical displacement signal by integrating the processed vertical velocity signal, shown in Figure 5(c). Then, we apply EMD again and retain the first few (we experimentally chose first three high-frequency components) IMF components to suppress the accumulation error.
4. Calculate the vertical distance *h* for each step by measuring the difference between the crest and trough, as shown in Figure 5(d).
5. Calculate the step length of each step using equation (12) with the parameter *h*, measured leg length *l*, and foot length *S*.

### Peak detection algorithm

**Figure 6:**
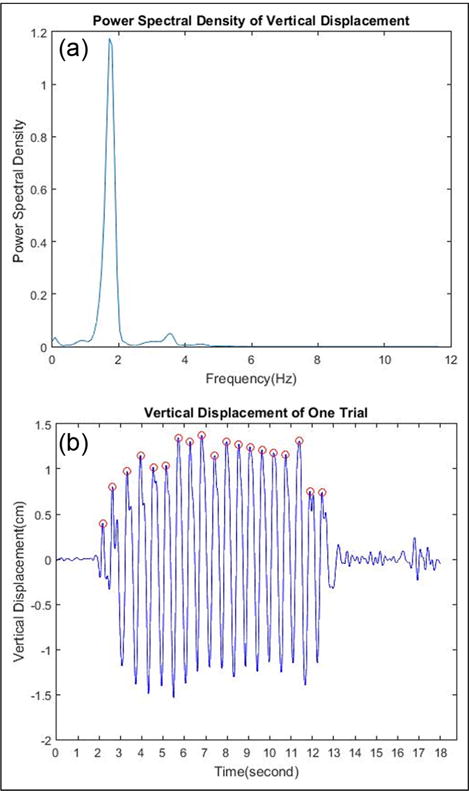
(a) Power spectral density of vertical displacement. (b) Peak detection results of one trial.

After the double integrations and EMD process, we get the vertical displacement signal without any drifting errors. We count steps using the vertical displacement signal by the following steps:

1. Calculate the power spectral density of this displacement signal, as shown in Figure 6(a). The walking frequency is determined at the peak value of the spectrum. The average number of sampling points of one step *P* is calculated by sampling frequency divided by walking frequency.
2. Find the first peak in the vertical displacement signal. If this peak is higher than the empirically determined threshold (empirically threshold defined about 0.2–0.4 times of maximum value), it is used to define the start of walking.
3. Find the next peak point within a certain range (Min, Max) away from the current peak point. We set Min to 0.7 P and Max to 1.3 P. If there are more than one peak in this range, choose the maximum value.
4. If the new peak value is larger than the threshold, count steps and make this peak to be the reference point. Then, repeat step 3.
5. If this value is not larger than the threshold, set the reference point to be the previous peak point added Min. Then, repeat step 3.
6. Each detected peak point represents one single step in a steady walking period. Figure 6(b) shows the result of peak detection of one trial.

After we marked the peaks of each trial, the step length will be calculated by the inverted pendulum model with equation (7).

## Experiments and results

### Experimental protocol

**Figure 7:**
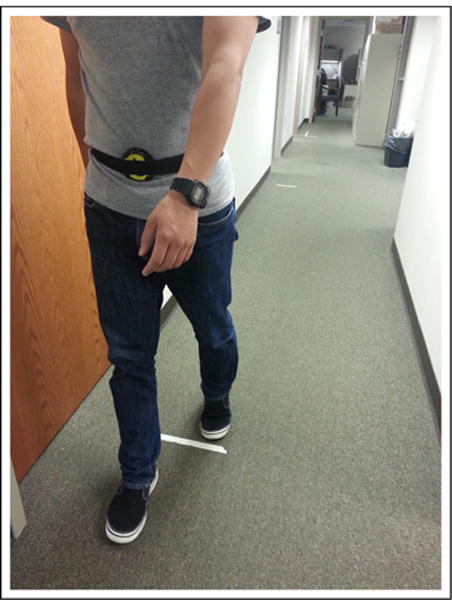
Subject walking on a 12-m walking pass with eButton strapped at lower abdomen.

A 12-m, straight-walking pass on a flat, hard-surfaced floor without obstacles nearby was selected in an indoor environment. A total of 10 healthy adult subjects participated in the study and walked through the pass wearing the eButton at the waist location, as shown in Figure 7. The test repeated three times at different walking speeds. The subjects received the following instructions in the beginning of the trials:

*Trial 1*. “Walk at your normal walking speed.”
*Trial 2*. “Walk as fast as you safely can, but do not run.”
*Trial 3*. “Walk slowly.”

It is important to mention that 12 m is a reference walking distance. In each trial, subjects were told to walk with their usual manner and would not be disturbed by the beginning position and ending position of the 12-m walking. So, the actual walking distance in each trial is around 12 m instead of strictly equal to 12 m. In this case, lab assistant recorded the real starting position and ending position of each trial to measure the real walking distance.

During each trial, our lab assistants counted the number of steps each subject walked. We use the reported count as the golden standard to compare with the computed result. The golden standard mean step length is calculated by actually walking distance divided by the step count.

The information of each subject, including leg length (from ground to waist) and foot length (from tiptoe to heel), is shown in Table 1. It shows the information of real walking distance, number of steps, and mean step length of each trail, which is calculated by walking distance divided by the step count.

### Find optimum value of K

**Figure 8:**
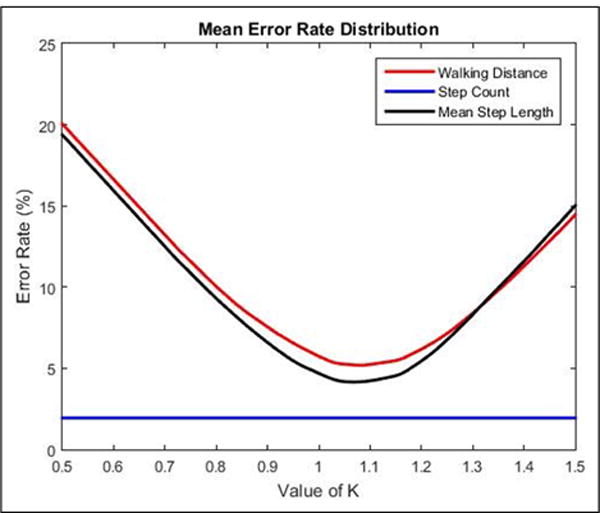
Mean error rate with different *K*.

Figure 8 shows the mean error rates of the walking distance, number of steps, and mean steps calculated by different *K*.

According to equation (7), we deduced the least squares optimization function

(16) $$R\left(K\right)={\sum_{i=1}^{10}\left\|\left(2\sqrt{2l_{i}-h_{i}^{2}}+K\cdot s_{i}\right)-SL_{\mathrm{real},i}\right\|}^{2}={\sum_{i=1}^{10}\left\|K\cdot s_{i}-(SL_{\mathrm{real},i}-2\sqrt{2l_{i}-h_{i}^{2}})\right\|}^{2}$$

where *l_i_* is the *i*th subject’s leg length, *h_i_* is the vertical displacement during one step of the *i*th subject, *s_i_* is the foot length of the *i*th subject, and *SL_real_*_,_ *_i_* is the real step length of the *i*th subject.

Then, the optimum value of *K* is given by

(17) $$\hat{K}={\left(S^{T}S\right)}^{-1}\cdot S^{T}\left(SL_{\mathrm{real}}-2\sqrt{2lh-h^{2}}\right)$$

where *S* = [*s*_1_*s*_2_ ⋯ *s*_10_]*^T^, l* = [*l*_1_*l*_2_ ⋯ *l*_10_]*^T^*, *h* = [*h*_1_*h*_2_ ⋯ *h*_10_]*^T^*, and *SL_real_* = [*SL_real_*_, 1_ *SL_real_*_, 2_ ⋯ *SL_real_*_, 10_]*^T^*

Through equation (17), we got the best *K* = 1.07, in equation (10).

### Results

Table 2 demonstrates part of the estimated result of each trial. The estimated walking distance is the sum of each step length, which is calculated by equation (10). The mean estimated step length is the quotient of the estimated walking distance divided by the estimated number of steps. Furthermore, we calculated the error rate of each trial by equation (18)

(18) $$\text{Error}\;\text{rate}=\left|\frac{\text{Actual}\;\text{value}-\text{Estimated}\;\text{value}}{\text{Actual}\;\text{value}}\right|\times 100\%$$

**Figure 9:**
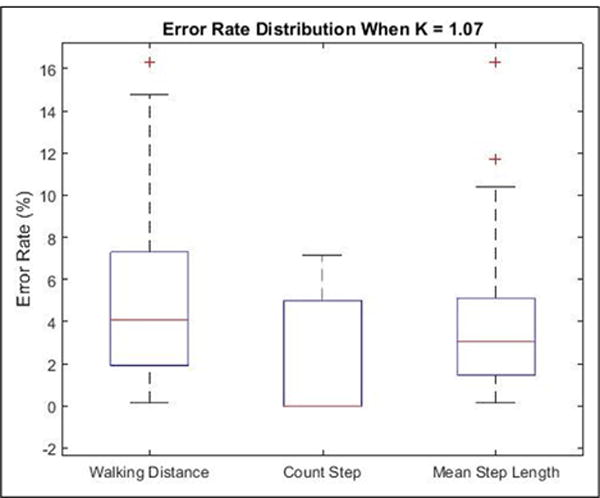
Box plot of error rate distribution of walking distance, number of steps, and mean step length. In each box, the central line represents the median error rate, and the bottom/top edges of the box are the first and third quartiles, and in the second box, the median error rate and the first quartiles error rate are both equal to zero. The outliers are plotted individually as plus sigh (+).

Figure 9 shows the box plot of the error rate distribution of walking distance, number of steps, and mean step length. The median error rates are below 5%, and the third quartiles of error rate is below 8%, which are acceptable in our study.

**Figure 10:**
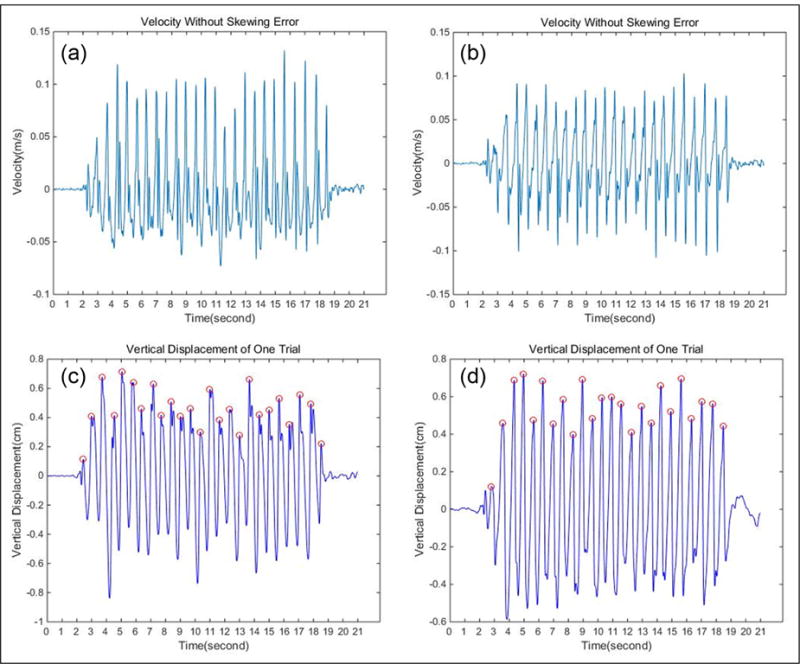
Different performances between EMD and the high-pass filter: (a) vertical velocity signal after EMD, (b) vertical velocity signal after a high-pass filter, (c) peak detection result after EMD, and (d) peak detection result after a high-pass filter.

To validate the effectiveness of our EMD algorithm, we estimated the walking distance, number of steps, and mean step length using the same experiment data in different speed with a high-pass filter. Figure 10 shows the different performances between EMD and the high-pass filter. Figure 10(a) shows the vertical velocity signal after EMD, and the original signal is shown in Figure 5(a) which contains accumulation error. Figure 10(b) shows the vertical velocity signal after a high-pass filter with the same original signal.

Table 3 shows the error rates calculated by the method with EMD and the method with a high-pass filter. According to the table, our method with EMD has higher accuracy than a high-pass filter. In addition, the method with EMD is much more adaptive, because when we use the high-pass filter to reduce the mean trend or drift, a cutoff frequency must be selected which depends on different people and different walking speeds. But when we use the EMD approach, no additional parameters need to be selected, and the mean trend or drift of any trial can be eliminated by subtracting the last two IMF components. Therefore, compared with the high-pass filter, our EMD method is more accurate, adaptive, and robust.

The coordinate transformation DCM is one of the most important improvements in our approach. Table 4 shows the difference in mean error rates between the method with DCM + EMD and the method with EMD only. It can be seen that the data processing method with DCM for coordinate transformation improves the accuracy of the step count and mean step length estimation, while it has few influence on walking distance estimation.

The error rate of the mean step length estimation is reduced by the DCM algorithm in all speed situations, which is shown in Table 4. Furthermore, the standard error rate reduction is calculated by equation (19), where *N* represents the number of subjects, *ER_DCM_* represents the error rate with DCM + EMD, and *ER_UDCM_* represents the error rate with EMD only. First, we calculate the reduction of each subject’s estimated step length error rate between the method with DCM + EMD and the method with EMD only in different speed situations. Then, the standard error rate reduction, which is shown in Table 5, is calculated by the standard deviation of five subjects’ error rate reductions. The standard error rate reduction is −1.69% in the slow speed, while the result is −1.95% in the normal speed and −3.56% in the fast speed. So, obviously, the effectiveness of the DCM algorithm has been improved while the walking speed increased

(19) $$\text{Standard}\;\text{error}\;\text{reduction}=-\sqrt{\frac{\sum_{i=1}^{N}{\left(ER_{\mathrm{DCM}}-ER_{U\mathrm{DCM}}\right)}^{2}}{N}}\times 100\%$$

**Figure 11:**
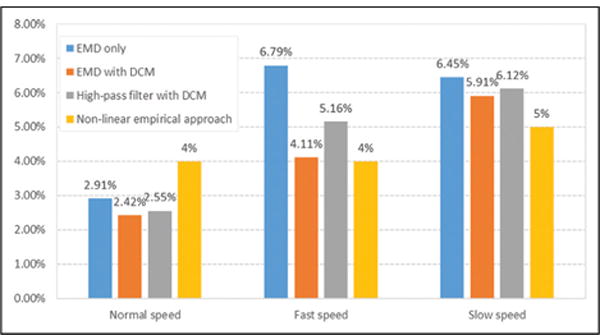
Mean error rates of different step length estimation methods.

Figure 11 shows the mean error rates of different step length estimation methods. To compare with the empirical method, we select the well-performed non-linear empirical approach proposed by S Shin.^9^ The mean error rate of the empirical approach is 4% in normal and fast speed and 5% in slow speed as shown in Figure 11. In addition, the mean error rate of another empirical method proposed by V Renaudin^7^ could reach 5%. It can be seen that our method with EMD and DCM got the best performance in normal speed and as good as the non-linear empirical approach in fast speed. Otherwise, our method is much more adaptive than the empirical approach, while the latter should be trained again when the subject changed.

## Conclusion

This article presents a new data processing method to count steps and estimate the step length based on inverted pendulum model. A coordinate transformation is used to eliminate the orientation error due to the variations in wearing a motion sensor. The EMD is used to suppress the accumulation error caused by the integration procedure. Our method performed well in our experiments, which involves three different walking speeds.

There are three main differences between our method and previous pendulum model-based method:

1. We add coordinate transformation (DCM) to obtain vertical acceleration after median and low-pass filtering in data preprocessing.
2. We use the EMD to eliminate the accumulation error due to integration.
3. Unlike previous studies which focused on finding a right integrating range in each walking step, we integrate data as a whole without piecewise integrations.

According to the experimental results, our data process method with EMD and coordinate transformation could reach 97.58% accuracy in step length estimation, which is better than the high-pass filter approach or non-linear empirical approach in the same situation. Furthermore, the analysis of error rates’ reduction shows that the effectiveness of coordinate transformation will be improved while the walking speed increased. In addition, our method with EMD and coordinate transformation needs no more additional parameters, while the high-pass filter approach needs a cutoff frequency which depends on different people and different walking speeds and the empirical approach should be trained again when the subject changed. Therefore, our approach is more adaptive, robust, and accurate.

There are also some limitations in our method. First, the sensors have to be fixed on the trunk to ensure that the accelerations from sensors are similar to the accelerations of the body’s weight center. Second, the step length could be well estimated by this method in a continuous and relatively stable walking period, but the method is not applicable in the single step test or stop-and-go trips.

In the future, we would like to research on more situations like noise and interrupt, and we have to consider the various walking cases such as on stairs and steep roads to complete more adaptive step length estimation algorithms.

## Figures and Tables

**Table 1:** Experiment information of each trial and each subject.

No. of subjects	Length (cm)		No. of steps			Step length (cm)			Walking distance (m)		
	Leg	Foot	Normal	Fast	Slow	Normal	Fast	Slow	Normal	Fast	Slow
1	101.6	25.0	17	15	18	72.12	82.13	68.16	12.27	12.32	12.27
2	99.10	26.5	16	14	19	77.00	88.72	65.10	12.32	12.42	12.37
3	106.7	27.0	18	14	21	68.44	90.35	59.39	12.32	12.65	12.47
4	94.00	25.5	17	14	19	73.96	88.36	66.31	12.57	12.37	12.60
5	101.6	26.5	18	15	19	70.13	83.99	65.37	12.62	12.60	12.42
6	109.2	27.0	21	16	24	59.39	77.31	51.44	12.47	12.37	12.34
7	106.7	26.0	20	18	22	61.47	69.29	56.34	12.29	12.47	12.40
8	99.10	25.5	19	17	20	65.77	71.87	62.36	12.50	12.22	12.47
9	99.10	25.5	20	19	25	62.23	65.77	49.99	12.45	12.50	12.50
10	106.7	26.5	19	15	24	65.77	84.67	51.44	12.50	12.70	12.34

**Table 2:** Step length estimation result.

Test	Step counting		Step length (cm)			Distance (m)		
	Ref.	Estimated	Ref.	Estimated	Error (%)	Ref.	Estimated	Error (%)
*Normal*								
Sub1	17	16	72.12	72.57	0.62	12.27	11.61	5.37
Sub2	16	15	77.00	76.14	1.11	12.32	11.42	7.30
Sub3	18	18	68.44	71.27	4.13	12.32	12.82	4.05
Sub4	17	17	73.96	70.34	4.89	12.57	11.95	4.93
Sub5	18	18	70.13	70.91	1.11	12.62	12.76	1.10
*Fast*								
Sub1	15	15	82.13	77.44	5.71	12.32	11.61	5.76
Sub2	14	13	88.72	86.69	2.28	12.42	11.27	9.25
Sub3	14	14	90.35	86.73	4.00	12.65	12.14	4.03
Sub4	14	13	88.36	81.11	8.20	12.37	10.54	14.7
Sub5	15	15	83.99	81.71	2.71	12.60	12.25	2.77
*Slow*								
Sub1	18	18	68.16	66.10	3.02	12.27	11.89	3.09
Sub2	19	19	65.10	66.36	1.93	12.37	12.60	1.85
Sub3	21	21	59.39	66.33	11.6	12.47	13.93	11.7
Sub4	19	19	66.31	64.42	2.85	12.60	12.24	2.85
Sub5	19	19	65.37	66.33	1.46	12.42	12.60	1.44

**Table 3:** Mean error rates of the data processing method with EMD and high-pass filter.

Category	Data process method with EMD			Data process method with high-pass filter		
	Normal (%)	Fast (%)	Slow (%)	Normal (%)	Fast (%)	Slow (%)
Walking distance	4.39	5.65	5.55	4.38	4.69	5.85
Count step	2.18	3.19	0.45	2.23	4.39	4.20
Mean step length	2.42	4.11	5.91	2.55	5.16	6.12

EMD: empirical mode decomposition.

**Table 4:** Mean error rates of the data processing method with DCM + EMD and EMD only.

Category	Data process method with EMD only			Data process method with DCM + EMD		
	Normal (%)	Fast (%)	Slow (%)	Normal (%)	Fast (%)	Slow (%)
Walking distance	5.10	5.97	4.41	4.39	5.65	5.55
Count step	4.04	3.58	3.23	2.18	3.19	0.45
Mean step length	2.91	6.79	6.45	2.42	4.11	5.91

EMD: empirical mode decomposition; DCM: direction cosine matrix.

**Table 5:** Standard error reduction of the method with DCM + EMD and EMD only in different speeds.

No. of subjects	Slow speed error (%)		Normal speed error (%)		Fast speed error (%)	
	EMD only	DCM + EMD	EMD only	DCM + EMD	EMD only	DCM + EMD
Sub1	5.58	3.03	1.99	0.55	9.48	5.71
Sub2	0.32	1.92	4.55	1.11	7.91	2.28
Sub3	10.7	11.7	2.21	4.13	6.39	4.00
Sub4	4.05	2.85	5.98	4.86	10.3	8.20
Sub5	3.15	1.46	0.66	1.14	5.43	2.72
Standard error reduction	−1.69%		−1.95%		−3.56%	

EMD: empirical mode decomposition; DCM: direction cosine matrix.
